# Balancing principles, power, and pragmatism: reflections on co-production in establishing national research priorities for women’s sport and physical activity

**DOI:** 10.1093/heapro/daag159

**Published:** 2026-11-04

**Authors:** Emma S Cowley, Denise Martin, Jennifer Murphy, Aoife Lane, Eimear Kelly, Maria Faulkner, Maria O’Kane, Angela Carlin, Mary Margaret Meade, Elizabeth Deery, Patricia C Jackman

**Affiliations:** SHE Research Centre, Department of Sport and Health Sciences, Technological University of the Shannon, Dublin Road, Athlone, Co. Westmeath N37 HD68, Ireland; Department of Sport, Exercise and Nutrition, Atlantic Technological University, Dublin Road, Galway City H91 T8NW, Ireland; Department of Sport Science and Performance, Atlantic Technological University, Donegal Letterkenny, Port Road, Letterkenny, Co. Donegal F92 FC93, Ireland; Department of Sport, Exercise and Nutrition, Atlantic Technological University, Dublin Road, Galway City H91 T8NW, Ireland; SHE Research Centre, Department of Sport and Health Sciences, Technological University of the Shannon, Dublin Road, Athlone, Co. Westmeath N37 HD68, Ireland; Department of Sport, Exercise and Nutrition, Atlantic Technological University, Dublin Road, Galway City H91 T8NW, Ireland; Department of Sport, Leadership & Human Performance, Technological University of the Shannon, Midwest, Moylish Park, Limerick V94 EC5T, Ireland; Department of Sport Science and Performance, Atlantic Technological University, Donegal Letterkenny, Port Road, Letterkenny, Co. Donegal F92 FC93, Ireland; Institute of Nursing and Health Research, Ulster University, Northland Road, L'Derry BT58 7JL, Northern Ireland; Centre for Exercise Medicine, Physical Activity and Health, Sports and Exercise Sciences Research Institute, Ulster University, Belfast BT15 1ED, United Kingdom; Centre for Exercise Medicine, Physical Activity and Health, Sports and Exercise Sciences Research Institute, Ulster University, Belfast BT15 1ED, United Kingdom; Centre for Exercise Medicine, Physical Activity and Health, Sports and Exercise Sciences Research Institute, Ulster University, Belfast BT15 1ED, United Kingdom; School of Psychology, Sport Science and Wellbeing, University of Lincoln, Brayford Pool, Lincoln LN6 7TS, United Kingdom

**Keywords:** participatory methods, stakeholder engagement, public involvement, knowledge translation, research governance, methodological reflexivity, interdisciplinary, health policy

## Abstract

Interest in co-production within sport, exercise, and health sciences has grown in recent years. The use of this approach, however, is not without challenges. In this article, we share reflections on a co-produced project that sought to establish research priorities for women in physical activity, sport, and exercise on the island of Ireland. More specifically, we analyzed reflections from 17 public contributors—known as the Research Collective—and an interdisciplinary research team of 10 academics who collaborated to develop a survey, distributed nationally, to establish the research priorities. Our reflections highlighted persistent tensions between our ability to deliver our intended co-production approach while operating within structural constraints, including limited funding, institutional bureaucracy, and a short funding timeframe. We also identified challenges with relationship building and diversity, such as sustaining engagement beyond the project timeframe and ensuring representation of marginalized sub-groups. For the research team, these tensions and the need to compromise our approach carried an emotional weight that both shaped and was shaped by the (re)negotiation of power with the Research Collective. To support the advancement of co-production practice, we conclude by offering recommendations for researchers based on our learnings from this project.

## Graphical Abstract

**Graphical Abstract:**
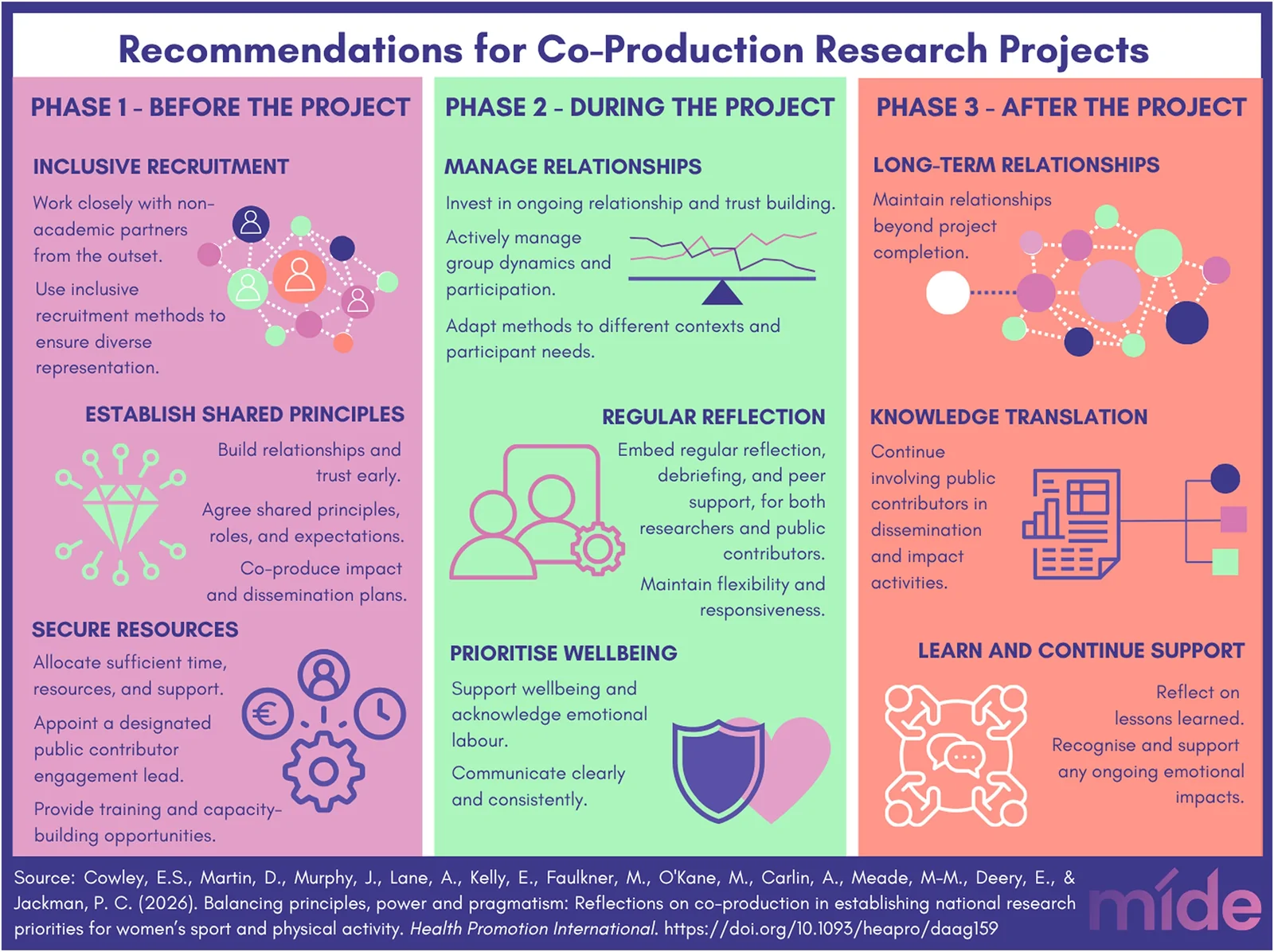
Graphical abstract for Balancing principles, power and pragmatism: Reflections on co-production in establishing national research priorities for women’s sport and physical activity.

Contribution to Health Promotion

- This article provides reflections on a co-produced project that sought to establish research priorities for women in physical activity, sport, and exercise on the island of Ireland.
- The complexities of sharing power in co-produced research are highlighted.
- Lessons learned from the project to support researchers with designing inclusive and equitable co-production research are outlined.

## Introduction

Over the last decade, there have been increasing calls from research organizations, funding bodies, and policymakers to move away from doing research *on* participants and instead to work *with* them, by actively involving citizens, non-academic partners, and communities as collaborators to ‘co-produce’ research (Canadian Institutes of Health Research 2014, van der Graaf *et al*. 2021). Co-production is a contested concept and is often used interchangeably with terms such as collaboration, consultation, and participatory research depending on the context and discipline (Smith *et al*. 2023b). In this article, co-production refers to approaches that seek to dissolve traditional boundaries between researchers and public contributors by recognizing multiple forms of expertise as equally valuable (British Academy 2008).

Co-production has been adopted to achieve a variety of outcomes, including intervention and service development (Buckley *et al*. 2022), resource development (Wadley *et al*. 2026), policy formation (Van Damme *et al*. 2016), and research priority-setting activities (Ikhile *et al*. 2023). While the goals and processes of co-production can differ depending on the typology being used (Smith *et al*. 2023b), co-produced research seeks to challenge traditional power hierarchies in research by emphasizing the importance of equitable partnerships between academics and community partners with relevant lived experience and/or experiential knowledge. Co-production thus builds upon traditions of participatory action research (Lewin 1946) and knowledge exchange (Beal *et al*. 1986) and is often used with the aim of increasing research impact (Smith and McGannon 2023) and reducing the research-to-practice gap (Durose *et al*. 2012). Beyond its instrumental value, many scholars also argue that involving those most affected by research is a moral and ethical imperative (Smith *et al*. 2023b).

A core principle of co-production is that people with lived experience of the issues being researched hold valuable insights and forms of expertise that can enhance the production of knowledge (British Academy 2008). Reflecting this principle, it is increasingly recognized that diverse experiences and knowledge from public contributors, including practitioners and community members, are essential to ensuring that interventions, services, and policies are contextually appropriate, impactful, and socially robust (Turnhout *et al*. 2020). Evidence suggests that meaningfully engaging with public contributors in co-produced research can generate a wide range of tangible benefits, including enhanced intervention relevance and effectiveness (O'Cathain *et al*. 2019), improved user experiences and safer services (Jennings and Szekeres 2025), and better overall intervention and service outcomes (Atkin *et al*. 2020). The value of co-production, therefore, can extend beyond individual research projects, contributing to the uptake, scalability, and sustainability of interventions, services, and policies in real-world settings (Dingelstad *et al*. 2025). Thus, co-production is not simply a methodological approach for enhancing research quality but a means of facilitating meaningful social change and improved real-world outcomes (Flinders *et al*. 2016, Williams *et al*. 2026).

Despite its transformative potential, co-production also presents substantial challenges that warrant careful consideration (e.g. Finders *et al*. 2016, Turnhout *et al*. 2020, Williams *et al*. 2020, Williams *et al*. 2026). Numerous logistical challenges can impede successful implementation, including its time-intensive nature and ethical complexity. Rigid academic structures and short funding cycles pose significant barriers given the substantial resources and capacity co-production requires. Because co-production relies on relationships where trust and rapport are built over time, it also carries relational vulnerabilities and challenges (Smith *et al*. 2023b). Sustaining this level of commitment can be difficult in practice, particularly as public contributors often have competing demands and priorities (e.g. employment, caring responsibilities). Furthermore, if involvement becomes tokenistic, public contributors may be included symbolically rather than substantively, limiting their capacity to shape decisions and potentially reproducing existing power imbalances within research (Egid *et al*. 2021).

There is also ongoing debate regarding the legitimacy of co-production as a research approach, with questions asked about the robustness of its outputs, particularly within more traditional or positivist academic communities (Flinders *et al*. 2016). Some have even suggested that co-produced research may represent a professional risk, as many researchers engage in these approaches with limited prior experience, meaning they must learn new skills while simultaneously challenging traditional academic norms, often facing resistance from peers, reviewers, and institutions (Flinders *et al*. 2016). Furthermore, concerns have also been raised about the extent to which the growing momentum behind co-production may be connected to academic audit cultures rather than genuine interest in engaging with community partners (Hiemstra and Matthews 2025), leaving co-production at risk of becoming a performative buzzword (Smith *et al*. 2023b). Given these challenges and the proliferation of interest in co-production, critical reflection is essential for understanding, interrogating, learning from, and improving co-production practice.

### Reflections on co-production research in sport and exercise

The use of co-production has grown within sport and exercise science, with many exemplars included in a recent special issue on co-design, co-production, and community-based research in *Qualitative Research in Sport, Exercise, and Health* (Smith *et al*. 2023a). In applied research, reflective practice is considered a core principle whereby behaviours and experiences are reflected upon, either in written or spoken form, to consolidate learning and improve future practice (Macaulay and Winyard 2012). Within co-production, reflection is not only a methodological tool but also an ethical responsibility (Page 2022). Reflection encourages exploration of both a project’s successes and shortcomings by asking questions about what went well, what challenges emerged, and why key events unfolded as they did. Approaching such reflections critically and reflexively enables ongoing learning, adaptation, and transparency, especially in terms of how power and relationships are managed (Koshy *et al*. 2017). However, despite calls for reflections on co-production in sport and exercise, exemplars remain scarce, with only a few emerging to date (e.g. Pettican *et al*. 2023 ; Williams *et al*. 2026). This scarcity may partly reflect concerns about academic credibility or perceived risks of critique for not achieving indicators of ‘gold-standard’ co-production, even when intended. Consequently, idealized representations of co-production may continue to dominate, leaving researchers ill-prepared for the messy and complex realities of implementation. As a result, researchers have been encouraged to present transparent, reflexive accounts of co-production, documenting both the aspirations and realities of co-production in practice (Pettican *et al*. 2023 ; Smith *et al*. 2023b, Williams *et al*. 2026).

### The present article

The aim of this article is to present a critical reflection from members of an interdisciplinary research team that co-produced a study seeking to identify research priorities for women in physical activity (i.e. physical activity, sport, and exercise) on the island of Ireland (míde research 2025). Drawing on multiple perspectives from both the research team and a public contributor group, we highlight the possibilities and pragmatic constraints of co-production by describing our process and reflecting critically on our experiences. By doing so, we seek to contribute fresh insights into the realities of co-production that can support the continued advancement of this approach across different fields.

## Methodology

### Background to the project

míde is an interdisciplinary research consortium based on the island of Ireland established in 2025 to undertake and disseminate research about women in physical activity, sport, and exercise. Since its formation, míde has instigated a number of collaborative research initiatives across higher education institutes on the island (Lane *et al*. 2026). A foundational project was a scoping review of existing research, which highlighted research trends and gaps relating to women in sport on the island of Ireland (Jackman *et al*. 2025). A key finding was the lack of involvement of non-academic partners in research conducted to date, with only 3.6% of studies reporting any form of engagement. To address this gap, an interdisciplinary team of 10 researchers from míde research partnered with two local sport partnerships (i.e. county-level organizations in the Republic of Ireland aiming to promote physical activity) and successfully applied for funding (€18 000) from Sport Ireland to undertake a 12-month research project aiming to establish priorities for research about women in physical activity across the island of Ireland. Critically, we sought to ensure that the research agenda was shaped by potential real-world beneficiaries rather than driven solely by academics, making it responsive to the experiences, needs, and preferences of those involved in women’s physical activity across the island.

### Philosophical positioning

Our research was underpinned by interpretivism, whereby we assumed reality and truth are multiple and context-dependent (i.e. ontological relativism) and that knowledge is subjective and socially constructed (i.e. transactional epistemology) (Smith and Sparkes 2016). Aligned with this philosophical perspective, we recognized that the production of knowledge is shaped by power relations and unequal social structures. Research agendas, for example, are not neutral but reflect existing power relations, with researchers, funders, and academic institutions traditionally exerting considerable influence over which questions are considered worthy of investigation and thus receive attention and resources. We approached co-production as a process through which academic and non-academic partners could collectively challenge these traditional power imbalances and equitably co-create knowledge (Monforte *et al*. 2022). Drawing on a relational perspective on power/knowledge (Foucault 1982), we understood co-production as more than a shifting of power relations between academics and public contributors. Rather, we viewed it as a dynamic and relational process through which traditional assumptions about who holds expertise and whose knowledge matters in research could be questioned and re-negotiated. By involving public contributors in establishing the research priorities, we sought to challenge these established power/knowledge relations and create space for those most likely to use and be impacted by the research to shape the future direction of research about women in physical activity on the island of Ireland.

### Co-production approach

Regarding the type of co-production chosen (see Smith *et al*. 2023b), we sought to deliver an equitable and experientially informed co-produced research project. We recognized that the lived experiences and experiential knowledge of those involved in women’s physical activity were essential to the research process. Our commitment to sharing power was evident from the outset, with our research team partnering with two Local Sports Partnerships to develop the initial funding application. The approach proposed comprised the establishment of a public contributor group, followed by two phases of fieldwork (i.e. a mixed-methods survey and qualitative interviews) for the priority-setting exercise, and finishing with the reporting and dissemination of findings. A key aspect of the research project was that we sought to collaborate with a diverse stakeholder group, the míde research collective (referred to hereafter as ‘the Collective’), to develop the survey and qualitative interview materials. Overall, we sought to embed co-production across multiple project phases, encompassing co-production of the research priority-setting project, methods used to establish the priorities, and identification of the future research priorities themselves. Based on the ladder of participation advanced by Arnstein *et al*. (1969), co-production of the research project was initially intended to be ‘partnership’ (rung 6), while the co-production of the research priorities sought to confer ‘citizen control’ (rung 8). Due to unexpected institutional delays (i.e. human resources, finance approvals) in the first three months of the project, we had to adapt our intended methods. As these delays occurred before the Collective was formed and compromised our ability to deliver the intended project within the required timeframe, researchers on the project team decided to solely deliver the survey phase of the proposed approach (something we expand upon further in our reflections). In this paper, we focus specifically on the process of working with the Collective during the project.

### Researcher positioning

The research team consisted of 10 women, all of whom were members of míde research, with expertise spanning different areas of sport and exercise science. Collectively, we brought different levels of experience in participatory research methods and co-production. Despite our formal role of ‘academics’ in the project, our own lived (and often challenging) experiences in sport and physical activity inevitably shaped our interest in, commitment to, and engagement with this research. In this sense, our involvement was informed not only by professional expertise but also experiential knowledge, for example, as athletes, coaches, exercisers, practitioners, and mothers of sport participants. We believed that co-producing a research agenda for women in physical activity on the island of Ireland had the potential to generate knowledge that was more relevant, meaningful, and actionable. This intersection of our personal and professional identities, alongside these moral foundations, meant that this project was much more than an intellectual endeavour, but one we deeply cared about and were emotionally attached to. Although we aspired to work equitably with our non-academic partners, we remained conscious that our academic positions brought a structural authority and provided access to institutional resources and research expertise that impacted the distribution of influence. Accepting that these forms of influence could not be removed, we realized that these dynamics necessitated ongoing attention and critical reflection. In practice, this meant being attentive to assumptions, methodological expertise, decision-making processes, interactions, and experiences, and remaining reflexive about power dynamics.

### Forming the research collective

Once the project had begun, multiple meetings took place between the researchers and the partnering Local Sports Partnerships to agree on the process for forming the Collective. Subsequently, an advertisement outlining the project aim and expected time commitment was circulated via social media (Supplementary Material S1), and a list of organizations and individual contacts who were asked to further share the opportunity across their networks was compiled. Sixty-three people (58 women; 92%) with experience of working with women in physical activity expressed interest in joining the Collective (Supplementary Material S2). Applicant names were anonymised by one author (DM) before three members of the research team (ESC, JM, PCJ), without extensive knowledge of the physical activity landscape on the island of Ireland, reviewed the expressions of interest independently. This process aimed to ensure equity and representation of people with experience of working across different categories of participants, including: disabled people, LGBTQI+ communities, varying age groups, different ethnic backgrounds, rural and urban settings, contrasting domains (i.e. physical activity and competitive sport), and an array of levels (e.g. grassroots and high performance). Two researchers (DM, AL) with extensive connections across the island’s physical activity and sport sectors then reviewed the 15 shortlisted candidates to sense-check whether any individuals or groups were underrepresented. Following this process, two additional participants were added from the original pool to address gaps. Table 1 provides an overview of demographic information for the 17-member Collective.

**Table 1:** Demographic information for the Research Collective members.

Category	Sub-category	*N* (%)
Gender	Woman	14 (82.4%)
	Man	3 (17.6%)
Ethnicity	White	17 (100.0%)
Location	Republic of Ireland	11 (64.7%)
	Northern Ireland	3 (17.6%)
	Republic of Ireland and Northern Ireland	3 (17.6%)
Role	Professional working in sport or exercise	13 (76.5%)
	Volunteer in sport or exercise	4 (23.5%)
	Academic in sport and exercise (in addition to professional or voluntary role)	3 (17.6%)
Groups worked with	Predominantly women/girls	9 (52.9%)
	Mixed gender groups	11 (64.7%)
	Young children	9 (52.9%)
	Adolescents	15 (88.2%)
	Adults	13 (76.5%)
	Older adults (65+ years)	5 (29.4%)
	Different ethnicity	5 (29.4%)
	LGBTQI+	5 (29.4%)
	Disabilities	10 (58.8%)
	Disadvantage (i.e. socioeconomic positioning)	7 (41.2%)

### Engagement between the project team and the Collective

A summary of tasks completed by the project team and the Collective is presented in Table 2. Two researchers (DM, JM), funded for two hours per week on the project, were responsible for project leadership and management. Prior to the first Collective workshop (February 2025), the research team met to develop a draft survey (January 2025). With project delays shortening the time available to develop the survey in partnership with the Collective, we adopted a pragmatic compromise, drafting an initial survey as a research team and asking the Collective to provide feedback on this. While this helped to maintain momentum, it represented an initial shift of power towards the research team. Drawing on items from the Irish Sport Monitor (Sport Ireland 2024) and findings from our previous scoping review (Jackman *et al*. 2025), the draft survey comprised three sections: participant background, knowledge generation (i.e. research priorities), and knowledge translation (i.e. engagement with and perceptions of research about women in physical activity).

**Table 2:** Summary of project timeline and tasks.

Time period	Research team (RT)	Research collective (RC)
October–December 2024	*Project Leads* Communicate with partners, RT, and RC Create RC recruitment advert and EoI form Communicate with RC *Research Team (7 meetings)* Adapt research methods (due to delays) Agree RC recruitment process Advertise for RC Screen EoIs and select RC	Complete expression of interest (EoI) Form (Dec 2 deadline) Invitation to join RC
January–February 2025	*Project Leads* Communicate with partners, RT, and RC Create/amend survey and RC feedback forms Submit ethics application *Research Team (4 meetings)* Draft survey questions and send to RC Plan and facilitate RC Workshop 1 Develop dissemination plan	Complete review of draft survey Participate in online RC Workshop 1 Provide feedback on survey questions and involvement in project
March–June 2025	*Project Leads* Communicate with partners, RT, and RC Create final survey Create feedback form for RC *Research Team (5 meetings)* Survey launch and data collection (May–June) Plan analysis Plan and facilitate RC Workshop 2	Support survey sharing Review preliminary survey data Participate in online RC Workshop 2 Provide feedback on preliminary data, coding, and involvement in project
July–October 2025	*Project Leads* Communicate with partners, RT, and RC Create final report and presentation assets *Research Team (2 meetings)* Preliminary analysis of data Share preliminary analysis with RC and incorporate feedback Finalize analysis and draft outputs Submit report to funder (Oct)	Provide feedback on preliminary report (Sept) Conclusion and invitation to míde conference and to participate in focus group
Beyond project timeframe	*Research Team (2 meetings)* Share findings at míde research conference (Feb) and international conferences (Jun–Jul) Prepare and submit academic outputs (2026)	Focus group at míde conference (*n* = 3) and subsequent interview (*n* = 1)

Abbreviations: EoI = expression of interest; RC = míde research collective; RT = research team. The RT consisted of two Project Leads, eight other researchers, and two partners (i.e. Local Sport Partnerships).

The first online Collective workshop (February 2025), attended by 15 Collective members and seven researchers, aimed to introduce the project, build relationships, establish working principles, and obtain the Collective’s feedback on the draft survey. Small-group discussions on the survey were facilitated through breakout rooms. The workshop was also recorded for absent members or later review. A follow-up email invited the Collective to provide additional comments and feedback on the workshop. After amendments to the survey, Collective members were invited to provide further feedback via two desk reviews prior to the survey launch. In total, 10 Collective members provided feedback (58.8%). Once launched, Collective members were asked to share the survey across their networks to aid recruitment.

The second online Collective workshop (June 2025), attended by 10 Collective members and six researchers, sought to provide an opportunity for reflection and discussion of the preliminary findings. The research team presented examples of their interpretations of the dataset across the three survey sections. The workshop followed a similar format to the first, whereby smaller group discussions were facilitated in breakout rooms and concluded with a summary discussion and identification of next steps. A follow-up email to the Collective members invited additional feedback via an online survey, with six members offering feedback (35.6%). The Collective’s interpretations helped shape which areas required more detailed examination and informed how the survey findings were framed within the final report. Meeting minutes circulated after both workshops detailed how the Collective members’ contributions informed the process.

In the final stages, the preliminary report was circulated to the Collective via email, and they were invited to provide feedback via an online survey. In addition, information about how the Collective could be remunerated for their participation was provided. After the report was finalized, Collective members were invited to participate in post-project face-to-face focus groups at the annual míde conference (February 2026), with registration and accommodation costs covered. However, only three of the 17 members (17.6%) attended. For those unable to attend, online interviews were offered, with one member participating.

### Reflection process

Structured reflection was embedded throughout the project using Gibbs’ reflective cycle (1988) as a guiding framework. After each meeting, research team members were invited to reflect through an online form. The prompts operationalized four phases of Gibbs’ (1988) cycle and included questions such as ‘Describe an aspect of the co-production process from our work that stood out to you. How did this make you feel about the project?’ (description and feeling), ‘What do you think is going well in our co-production process? Were there any challenges, and if so, what impact did they have?’ (evaluation and analysis), and ‘Based on your experience so far, is there anything you’d suggest we adjust in our approach to improve the process?’ (conclusion and action plan). All reflections related to the co-production and research processes rather than to research involving participants and were collected to support collaborative learning and process evaluation. Reflections were voluntary, anonymised, and completed in the researchers’ own time to minimize potential power dynamics. Consistent with National Institute for Health Research (NIHR 2025) guidance on involvement, engagement, and co-production activities, these reflections did not require separate research ethics approval. In total, 60.0% (6/10) of the researchers completed at least one reflection across the project timeframe. In addition, during the peer review process for this journal article, several research team members engaged in additional co-reflexivity discussions via email and online calls. This more temporally distant opportunity to reflect on the project resulted in new reflective insights, which were subsequently added to our earlier reflections for analysis.

The Collective were also invited to provide feedback on their experience of the co-production process. Following each online workshop, Collective members were invited to provide anonymous feedback through an online form. The form requested information relating to their experiences of the workshops, opportunities to contribute, and whether they felt their views had been incorporated into the methods or outputs. Post-workshop evaluations were completed by 41.2% (7/17) and 35.3% (6/17) of Collective members after workshop 1 and workshop 2, respectively.

### Data analysis

Analysis of the reflections was initially led by the first author, with analysis led by the last author at a later stage, as we detail below. In the initial stage, the first author familiarized herself with the dataset (i.e. survey responses) and used the working principles for co-production proposed by Smith *et al*. (2023b) as a deductive framework to group extracts together. Accordingly, the six principles drawn upon were as follows: (1) co-production is adequately resourced; (2) power is shared through equitable partnerships; (3) different knowledge bases and contributions are valued; (4) relationships are based on mutual respect and dignity; (5) diversity is important and supported; and (6) reciprocity and mutuality are practiced. In addition, elements of a framework on the limitations and risks of co-production (Flinders *et al*. 2016) were also used as an interpretative lens. Drawing upon these frameworks simultaneously enabled us to examine structural barriers and institutional constraints that influenced our ability to uphold the principles for co-production in practice. The additional reflections generated during the peer review process were analysed by the last author and generated new interpretations, especially related to the complexities of power sharing and emotional aspects of co-production, which we subsequently integrated into our write-up. While the first and last authors led the analysis processes, other research team members engaged as critical friends (Smith and McGannon 2018), offering insights that encouraged the first and last authors to further reflect on and extend their analysis and how it was presented.

### Reflections

We structure our reflections sequentially in line with the six principles for co-production proposed by Smith *et al*. (2023b) but have selected labels for each section that represent our reflections. To ensure that an authentic and honest account was presented, rather than an idealized perspective, we have deliberately sought to preserve the contradictions, tensions, partial successes, and shortcomings of our project, with illustrative extracts from the research team and Collective members presented throughout.

### Tensions between ambition, co-production ideals, and resource realities

For meaningful collaboration, co-production requires adequate resourcing with sufficient time and funding (Smith *et al*. 2023b). However, researchers often face pragmatic constraints, including insufficient budgets, short timelines, and institutional bureaucracy, requiring what some have termed ‘satisficing’ or acceptance of less-than-ideal solutions to meet project constraints (Flinders *et al*. 2016). This tension between ideals and practical realities emerged early in our project, when it became clear that it was not feasible to deliver our intended co-production approach within the project timeframe. The delayed hiring of a research assistant slowed progress, and tasks had to be redistributed among the research team. However, we relied heavily on in-kind contributions, with time for only two of the 10 researchers funded by the grant. This made committing time to the project difficult, as one researcher explained: ‘This work is generally over and above our already busy schedules, so it is challenging to get everyone to meetings’. Even for the two members of our team whose time was funded, the time required exceeded what was allocated:

The reality is that the two hours [release from teaching] was way too little compared to the volume of work that needed to be done in the week. I ended up putting in far more time than the two hours but always with the feeling of being totally overwhelmed by the project. I never felt I had the time to put into it in a way that felt like I was doing a good job.

As portrayed above, the mismatch between project demands and our capacity to meet them was not only problematic for project delivery but also incurred an emotional toll, evoking feelings of frustration, worry, and concern about our ability to remain faithful to our co-production plans and commitment to equitable partnership working.

While the external constraints undoubtedly contributed to these capacity issues, on reflection, we also realize that we did, at least in part, have some role in creating these challenges ourselves. In seeking to develop a competitive funding bid, we designed a project of significant scale, both in terms of our methodological approach and intended impacts. However, in retrospect, our proposal was arguably too ambitious in scope and exceeded the resources, timescale, and flexibility available in this funding call. While our ambition reflected a genuine desire to engage in meaningful co-production, it also contributed to challenges with workload, deliverability, and our ability to realize our co-production aspirations.

### Balancing progress, power, and partnerships

The second principle proposed by Smith *et al*. (2023b) emphasizes the importance of sharing power through equitable partnerships. Our ability to collaborate equally with public contributors was constrained by structural factors from the outset. As is common in academic research, the grant application was written prior to the project’s formal commencement, with the applicant team comprising 10 academic researchers and two representatives from Local Sports Partnerships. Consequently, the Collective had no input into the overarching research questions, budget allocation, time commitment, or meeting structure. On reflection, these pre-determined parameters, along with the heavy academic weighting of the grant applicant team, most of whom attended project meetings, may have impacted engagement, understanding of the project, and the sense of ownership among the Collective members. However, involving more public contributors—beyond the Local Sports Partnerships—before submission would have required significant time for recruitment and remuneration of participants, something that was not feasible without funding.

The need to deliver our project within pre-determined parameters reflects a fundamental tension within co-production identified by Flinders *et al.* (2016): funders want clear, actionable research questions and predefined outcomes, yet co-production is inherently ‘messy’ and requires time to build meaningful relationships around often undefined problems. In our project, we found ourselves caught between competing pressures—needing to provide clarity for the funder on methods, milestones, and outputs, while simultaneously creating genuine opportunities for public contributors to influence the research direction. These competing demands did not sit easily with one another, especially as we became increasingly aware of the time pressures faced following the initial delays. With the first meeting of the Collective taking place three months after initially intended, uncertainty around methods to be used for the priority-setting exercise persisted for longer than expected. This need to make timely decisions and maintain project momentum, while remaining committed to ensuring that the Collective could meaningfully influence the process, created unease. As one researcher articulated, ‘It can be overwhelming. Not knowing what exactly the research tools will be [used] is a challenge in terms of planning’. The uncertainty and challenges of renegotiating power and responsibility were not only practical but also emotional, as we attempted to navigate the challenges of balancing institutional and funder expectations with a genuine commitment to collaborative decision-making.

Despite our commitment to involving the Collective in decisions, budget limitations restricted the level of engagement we could reasonably ask of members (i.e. based on recommended remuneration rates), curtailing their opportunity for influence and raising questions about how equally power was shared in the relationship. At times, the Collective’s engagement more closely resembled consultation rather than genuine co-production (Turnhout *et al*. 2020). While the Collective contributed to decisions made on the survey, final decisions about the overall survey design and direct distribution channels were made by the researchers. The consultative nature of our approach to developing the survey was noted by one Collective member: ‘There are probably different levels of co-production. It did feel like [we were] giving feedback on something that already existed, rather than the blank page, post-it note environment’. While feedback from the Collective resulted in redrafting of the survey on two occasions, some raised concerns about the length of the survey after it had been launched: ‘I thought it was very long. When I distributed it out, I was getting feedback from people on the ground saying, ‘this is really long’. Collectively, these reflections illustrate that although the Collective had genuine influence on survey development, key decisions could have benefited from further dialogue and additional piloting.

Despite our concerns about the degree of equity when working with the Collective, feedback from members suggested they felt involved in the process, with some noting that ‘It gave me the green light to feel that I have a voice’ and ‘It was quite open. It felt like you were giving your feedback and were able to influence’. While the shift towards more consultative forms of engagement at stages was not ideal, Collective members’ reflections suggested that our more consultative approach to engaging with the group was collaborative and meaningful and did not constitute what some have termed a ‘tokenistic’ form of citizen engagement (Arnstein 1969).

### Respect and value for different knowledge and contributions

Within co-production, different expertise, knowledge, skills and experiential bases should be valued (Smith *et al*. 2023b). Reflecting on our intention to respect different forms of expertise, the Collective included representatives from national governing bodies, charities, parents, athletes, policymakers, practitioners, and coaches. To create an environment where all types of knowledge could be expressed, we co-created shared working principles during the first meeting. The Collective was invited to add to or refine our suggestions, which included *share the space* (everyone gets a chance to speak), *respect all opinions* (value diverse perspectives even if you disagree), and *stay present* (avoid multitasking). While these principles did align with some of the co-production principles outlined by Smith *et al*. (2023b), on reflection, we acknowledge that discussion of, and agreement on, working principles for the Collective would have benefited from more time, something we would advise others to do in future (see Williams *et al*. 2026).

Given the large number of Collective members, the use of breakout rooms was an important opportunity for everyone to share their thoughts. Collective members shared that the ‘breakout rooms for ideas and opinions were great’ and recommended that we ‘continue with the smaller breakout rooms for discussion’. Post-workshop written input also provided space for those less comfortable engaging verbally. Despite these efforts, limited meeting time (two 2-hour workshops, five months apart) and reliance on email interactions presented time-related challenges, with a recurring theme from Collective members being that more time would have been beneficial. As one member pointed out: ‘It would be nice to have longer discussion, however, I understand that time is precious and it is difficult to fit everything into the specified slot’. Concerns surrounding time were also shared by the research team: ‘[There was] Limited time to get through everything we needed to get through, so [I’m] unsure if everyone felt truly heard’. From a relational power perspective (Foucault 1982), these constraints illustrate how the production of knowledge was influenced by the temporal and organizational structures within which the project occurred.

Co-production outputs are often dismissed by academics as ‘light-weight’ or ‘lower quality’ (Williams *et al*. 2020, p. 5), risking rejection from high-impact journals (Turnhout *et al*. 2019). At the same time, Flinders *et al*. (2016) note that practitioner and policy partners often place greater value on community impact and actionable outputs over academic publications. In seeking to respect these different forms of knowledge and value, our first output was a freely accessible report, written with minimal jargon and supported by infographics (míde Research 2025). However, we acknowledge that the report may still have privileged academic knowledge and practices, for example, in the language used and presentation format (i.e. written report rather than more practical tools or resources). While we did not invite Collective members to contribute to the development of the report, their feedback on an early draft was requested. Overall, the feedback was very positive, with one Collective member stating: ‘[it is] Great to have this together in one space, presented in point form and very succinct. Really useful information to inform interventions, campaign and programmes going forward’. Despite the positive feedback on the eventual outputs produced, on reflection, we realized that the time pressures of our project prevented us from involving the Collective in developing the dissemination plan as we would have liked, something we would address in future research.

### The challenge of relational depth under constraints

Co-production requires genuine, consistent relationships sustained through trust, respect, and dignity (Smith *et al*. 2023b). Members of the Collective articulated the importance they placed on relationship-building, as one pointed out: ‘I always find that if you can get people where they can build relationships and get on, they’ll actually be more honest in their answers, they’ll be more transparent with the information they give you’. From the research team’s perspective, internal relationships were strong, supported by frequent contact and team meetings. In the early stages, communication with Collective members was consistent and regular updates were communicated. However, maintaining relationships with the Collective proved more challenging as the project evolved, as we struggled with timely communication while juggling Collective workshops, survey analysis, report writing, and end-of-semester teaching. Academic and grant timelines meant we prioritized outputs over relationship maintenance, risking that participants would feel their contributions were not valued. Less regular communication was noticed by some Collective members: ‘We were heavily involved from the initial stages until the questionnaire was done, and then there was no contact for a long, long time. Now I get that's probably because you're doing all the work’. While this Collective member appeared to appreciate that progress continued to be made, the challenges we encountered with maintaining communication—and therefore relationships—highlight how structural constraints and academic pressures can influence relational aspects of co-production in practice.

The limited number of online touchpoints in our time-constrained project also risked superficial engagement rather than the deep collaboration needed for meaningful co-production (Smith *et al*. 2023b). In retrospect, working with a large Collective on a comparatively small, time-bound project, with only two full-group meetings, made it difficult to manage differing perspectives and build the depth of relationships we were aiming for. With over 25 people involved across the research team and Collective, online workshops often prioritized sticking to a time-bound agenda and reaching agreement on decisions rather than creating space for slower, more relational conversations. While online meetings facilitated geographic reach, reduced the time and financial burden of travel, and supported sustainability efforts, some felt the experience may have been different in person. One Collective member questioned, ‘Would you get the same experience online compared to in person? Probably not’, while another noted that ‘Maybe more time and a face-to-face half or full day would be of value. Even hybrid style’. Despite these comments, only four Collective members availed of the funded opportunity to attend our in-person event, raising questions about how feasible in-person engagement would have been for a project of national scale.

### Practical challenges of achieving diversity in co-production

Co-production partnerships should embrace plurality of perspectives, particularly by involving people from marginalized groups whose voices are often excluded from research (Smith *et al*. 2023b). In recruiting for the Collective, we adopted an intersectional lens, recognizing that the experiences of those involved in women’s physical activity are shaped by multiple and intersecting systems of power and inequality (Sherry *et al*. 2024). Consistent with our commitment to challenging dominant knowledge hierarchies, we sought to include individuals from diverse backgrounds whose perspectives have often been marginalized or excluded from previous research in the region (Jackman *et al*. 2025). While our recruitment strategies sought to engage individuals from marginalized groups (e.g. non-White ethnic groups, disability, traveller community), including purposefully sending information about the Collective recruitment process to relevant organizations, a significant limitation of the recruitment process was that we failed to ask questions about applicants’ demographic information (e.g. ethnicity, age, disability). Although respondents were asked, ‘What groups did/do you primarily work with in women and girls’ sport?’, they were not asked to report on aspects of their own identity. As a result, we were unable to assess the diversity of the Collective or determine the extent to which its membership reflected the diversity of populations the project aimed to serve. Consequently, while Collective members brought valuable experience of working with a range of marginalized communities, they did not necessarily contribute the lived experiences associated with those identities.

When selecting members for the Collective, we sought to avoid recruiting the ‘usual suspects’ from existing networks (Smith *et al*. 2023b) by screening the expressions of interest independently and with identifiable information removed. However, senior researchers in the team retained final decision-making authority and added individuals they felt represented important missing voices. This decision revealed a tension between drawing on insider knowledge and avoiding overreliance on existing networks, reflecting the argument that researchers may struggle to recognize their own partiality (Smith *et al*. 2023b). One research team member reflected: ‘My sense is that the initial recruitment phase was biased to academics, researchers, large sporting bodies and perhaps did not find its way, was not perceived relevant, or feasible by some priority groups’. Although we contacted organizations representing marginalized groups in women’s physical activity, we did not achieve the diversity we aspired for. Recognizing the potential exclusion of important voices elicited a range of emotional responses for us as researchers, including ‘guilt and a degree of fear… what if we miss an important perspective?’ Together, these reflections demonstrate both the barriers created by our recruitment approach and the emotional labour involved in attempting to achieve diversity.

### Practicing reciprocity and mutuality

Co-production should be grounded in reciprocal partnerships in which all parties benefit from working together (Smith *et al*. 2023b), rather than one-directional relationships where academics are positioned as knowledge-holders and partners merely provide experiences or values to be corrected by academics (Turnhout *et al*. 2020). Reciprocity and mutuality were practiced relatively well within this project. From the research team’s perspective, the Collective provided valuable knowledge and experience that helped refine survey questions and facilitated access to diverse networks for survey dissemination. Their endorsement also strengthened the credibility and perceived relevance of the research.

Many Collective members reflected positively on the opportunity to learn through involvement, with one member stating: ‘It was a learning process for me. I learned loads’, while several also commented on feelings of excitement and enjoyment during the process. The project also brought people together from different sport organizations and sectors across the island of Ireland, creating opportunities for networking and future collaborations. One member reflected: ‘I was able to link in with people who I never would have thought of… all these people near me are doing fabulous work that weren’t even on my radar’. More tangibly, individual members were invited to review outputs from the project and were recognized as named authors on the final report (míde Research 2025). The Collective was also identified as a contributing co-author in academic outputs (e.g. Jackman *et al*. 2026).

## Conclusion and recommendations

In this reflective article, we documented our experiences of working with a public contributor group in a co-production project aiming to establish research priorities for women in physical activity across the island of Ireland. While we successfully created a Collective and produced accessible outputs, we experienced many challenges, and there were aspects of our work that fell short of ‘fully’ realizing principles for co-production (Smith *et al*. 2023b). In particular, our experiences reinforce the challenges of negotiating power in co-production (Williams *et al*. 2026) and provide an illustrative example of the complexities of power dynamics at multiple levels: power was not only enacted by individual decisions of the research team but by procedural and structural constraints that shaped the participation of public contributors and their opportunity to influence decision-making processes. Our reflective account also contributes insights on the affective realities of co-production, complementing recent perspectives in the field (Hiemstra and Matthews 2025).

Based on our reflections, we offer recommendations for researchers. Our experiences emphasize the importance of: working strongly with non-academic partners when co-producing funding applications; considering different recruitment methods for public contributors to ensure representation of marginalized sub-groups (e.g. combination of purposeful and open calls), including earlier engagement with community partners; embedding activities to build relationships and trust; co-producing a knowledge exchange and impact plan; having a designated public contributor engagement lead; being realistic about the time, resources, and adaptability needed for co-production; actively managing group dynamics (e.g. group size and interactions) and adapting approaches to different contexts (e.g. online vs. in person) and individual needs (e.g. communication and skills); explicitly agreeing on shared principles for co-production; providing training, support, or capacity-building needed to enable the involvement of lived experience contributors; being clear on expectations for public contributors, both during and beyond the project timeframe; and dedicating resources to post-project relationship maintenance. In addition, we encourage researchers to allow time for regular debriefs, reflection, and co-reflexivity, and to think creatively about different methods that could facilitate these, depending on the group, project stage, and setting. Including time, space, and funding to facilitate similar opportunities for public contributors is also recommended. Consideration, support, and compassion for the emotional impacts of co-production, both for researchers and non-academic partners, are also essential, especially when researching topics that could be distressing and/or emotionally laboursome.

Overall, the aim of our research project—to co-produce research priorities for women in physical activity on the island of Ireland—was delivered, but we encountered significant challenges when attempting to co-produce the methods for our priority-setting exercise. By honestly and transparently reporting our reflections, we hope this article provides insights that support others to engage in high-quality co-production. We encourage others to share honest reflections on both the aspirations and challenges of co-production, so the field can develop more grounded, sustained practices that reflect co-production’s complexities and dynamic realities.

## Supplementary Material

daag159_Supplementary_Data

## Data Availability

The data that support the findings of this study are available from the corresponding author upon reasonable request.

## References

[daag159-B1] Arnstein SR . A ladder of citizen participation. J Am Inst Plann 1969;35:216–24. 10.1080/01944366908977225

[daag159-B2] Atkin H , Thomson L, Wood O. Co-production in research: co-researcher perspectives on its value and challenges. Br J Occup Ther 2020;83:415–7. 10.1177/0308022620929542

[daag159-B3] Beal G , Dissanayake W, Konoshima S editors Knowledge Generation, Exchange, and Utilization. Boulder, CO: Westview Press, 1986.

[daag159-B4] British Academy . Punching our Weight. London: British Academy, 2008.

[daag159-B5] Buckley B , Newton J, Knox S et al Multi-stakeholder perspectives on co-production: five key recommendations following the Liverpool Co-PARS project. Qual Res Sport Exerc Health 2023;15:220–34. 10.1080/2159676X.2022.2152085

[daag159-B6] Canadian Institutes of Health Research . *Guide to knowledge translation planning at CIHR: integrated and end-of-grant approaches*. 2014 https://cihr-irsc.gc.ca/e/documents/kt_lm_ktplan-en.pdf (13 May 2026, date last accessed).

[daag159-B7] Dingelstad C , Hodyl N, Wyse R et al Co-production, co-creation and co-design in primary health care: a scoping review of design, implementation, impact and sustainability. Health Expect 2025;28:e70447. 10.1111/hex.70447 PMC12818046 41045116

[daag159-B8] Durose C , Beebeejaun Y, Rees J et al Towards Co-production in Research with Communities (Connected Communities). Arts and Humanities Research Council, 2012.

[daag159-B9] Egid BR , Roura M, Aktar B et al You want to deal with power while riding on power’: global perspectives on power in participatory health research and co-production approaches. BMJ Glob Health 2021;6:e006978. 10.1136/bmjgh-2021-006978 PMC8587355 34764147

[daag159-B10] Flinders M , Wood M, Cunningham M. The politics of co-production: risks, limits and pollution. Evid Policy 2016;12:261–79. 10.1332/174426415X14412037949967

[daag159-B11] Foucault M . The subject and power. Crit Inq 1982;8:777–95. 10.1086/448181

[daag159-B12] Gibbs G . Learning by Doing: a Guide to Teaching and Learning Methods. Oxford, UK: Oxford Further Education Unit, 1988.

[daag159-B13] Hiemstra M , Matthews CR. Feeling or funding? Critical coproduction, rationality, emotionality, and axiological reflections. Qual Inq 2025;31:542–54. 10.1177/10778004241265990

[daag159-B14] Ikhile D , Glass D, Frere-Smith K et al A virtuous cycle of co-production: reflections from a community priority-setting exercise. Health Expect 2023;26:2514–24. 10.1111/hex.13851 PMC10632611 37602918

[daag159-B15] Jackman P , Cowley E, Ní Chéilleachair N et al A scoping review of research about women in sport: a perspective from the island of Ireland. Perform Enhanc Health 2025;13:100335. 10.1016/j.peh.2025.100335

[daag159-B16] Jackman PC , Murphy J, Faulkner M et al Establishing research priorities for women’s physical activity, exercise, and sport on the island of Ireland: findings from the míde research collective. J Sports Sci 2026;44:33. 10.1080/02640414.2026.2668925

[daag159-B17] Jennings G , Szekeres Z. Towards co-production: the case of movement inspired by martial arts (MIMA). J Sport Soc Sci 2025;8:71–101. 10.6093/2611-6693/12872.

[daag159-B18] Koshy K , Limb C, Gundogan B et al Reflective practice in health care and how to reflect effectively. Int J Surg Oncol (N Y) 2017;2:20. 10.1097/ij9.0000000000000020 PMC5673148 29177215

[daag159-B19] Lane A , Martin D, Murphy N et al The míde research consortium: building a sustainable interdisciplinary community for research on women in sport, exercise, physical activity and health on the island of Ireland. Women Sport Phys Act J 2026;34:wspaj.2025-0143. 10.1123/wspaj.2025-0143

[daag159-B20] Lewin K . Action research and minority problems. Soc Issues 1946;2:34–46. 10.1111/j.1540-4560.1946.tb02295.x

[daag159-B21] Macaulay C , Winyard P. Reflection: tick box exercise or learning for all? Br Med J 2012;345:e7468. 10.1136/bmj.e7468

[daag159-B22] míde research (2025). *All-island research study: priorities for women’s physical activity, exercise and sport research on the island of Ireland*. míde Research. https://www.mideresearch.com/post/m%C3%ADde-research-collective-research-priorities-for-women-in-sport

[daag159-B23] Monforte J , Davis C, Saleem S et al Moving on from the Delphi study: the development of a physical activity training programme prototype through co-produced qualitative research. Qual Health Res 2022;32:1952–64. 10.1177/10497323221126535 PMC9629513 36315098

[daag159-B24] NIHR (2025). *A guide to co-production for researchers, services and commissioners*. Applied Research Collaboration Kent, Surrey and Sussex. https://arc-kss.nihr.ac.uk/document-download/77-what-is-co-production-april-2021/file [10 June 2026, date last accessed]

[daag159-B25] O'Cathain A , Croot L, Duncan E et al Guidance on how to develop complex interventions to improve health and healthcare. BMJ Open 2019;9:e029954. 10.1136/bmjopen-2019-029954 PMC6701588 31420394

[daag159-B26] Page K . Ethics and the co-production of knowledge. Public Health Res Pract 2022;32:e3222213. 10.17061/phrp3222213 35702747

[daag159-B27] Pettican A , Goodman B, Bryant W et al Doing together: reflections on facilitating the co-production of participatory action research with marginalised populations. Qual Res Sport Exer Health 2023;15:202–219. 10.1080/2159676X.2022.2146164

[daag159-B28] Sherry E , Bowell P, Symons K et al Researching women in sport development: an intersectional approach. Sport Soc 2024;27:820–42. 10.1080/17430437.2023.2278614

[daag159-B29] Smith B , McGannon K. Co-production and impact: challenges and opportunities. In: Denzin N, Lincoln Y, Giardina M, Cannella G (eds.), The Sage Handbook of Qualitative Research. Champaign, IL: SAGE, 2023, 535–48.

[daag159-B30] Smith B , McGannon K, Atkinson M. Advancing participatory approaches to co-production, co-design, and community based-research in sport, exercise and health: a curated collection of contemporary views. Qual Res Sport Exerc Health2023a;15. https://www.tandfonline.com/toc/rqrs21/15/2.

[daag159-B31] Smith B , Sparkes AC. Introduction: an invitation to qualitative research. In: Smith B, Sparkes AC (eds.), Routledge Handbook of Qualitative Research Methods in Sport and Exercise. Abingdon, OX: Routledge, 2016, 1–7.

[daag159-B32] Smith B , Williams O, Bone L. Co-production: a resource to guide co-producing research in the sport, exercise and health sciences. Qual Res Sport Exerc Health2023b;15:159–87. 10.1080/2159676X.2022.2052946

[daag159-B33] Sport Ireland . *Irish Sports Monitor 2024*. 2024. https://www.sportireland.ie/sites/default/files/media/document/2025-09/ISM%202024%20Summary%20Report%202024.pdf [accessed May 13, 2026]

[daag159-B34] Turnhout E , Metze T, Wyborn C et al The politics of co-production: participation, power, and transformation. Curr Opin Environ Sustain 2020;42:15–21. 10.1016/j.cosust.2019.11.009

[daag159-B35] Van Damme J , Caluwaerts D, Brans M. Coproduction in health planning: challenging the need for “open” policy-making processes. Int J Public Admin 2016;39:1056–66. 10.1080/01900692.2016.1186180

[daag159-B36] van der Graaf P , Cheethman M, Redgate S et al Co-production in local government: process, codification and capacity building of new knowledge in collective reflection spaces. Workshops findings from a UK mixed methods study. Health Res Policy Syst 2021;19:12. 10.1186/s12961-021-00677-2 PMC7844986 33514382

[daag159-B37] Wadley R , Okonkwo C, Roche K et al Co-produced research that bridges the ‘Knowledge Gap’ to support physical activity participation in disabled children and young people with limb difference. Qual Res Sport Exerc Health 2026;18:34–52. 10.1080/2159676X.2025.2534892

[daag159-B38] Williams O , Sarre P, Papoulias C et al Lost in the shadows: reflections on the dark side of co-production. Health Res Policy Syst 2020;18:43. 10.1186/s12961-020-00558-0 PMC7204208 32380998

[daag159-B39] Williams TL , Gabriel A, Falcon T et al Intersectionality and co-production in disability and physical activity research; critical reflections on promise, negotiation and transformation. Qual Res Sport Exerc Health 2026;18:414–32. 10.1080/2159676X.2026.2616516

