# Supplementary material for: Balancing principles, power, and pragmatism: reflections on co-production in establishing national research priorities for women’s sport and physical activity
Source: Health Promot Int. 2026 Nov 4;41(6):daag159. doi: 10.1093/heapro/daag159 (PMC13732305; doi:10.1093/heapro/daag159)
Supplement: Supplementary file 1 [file daag159_supplementary_data.zip › Supplementary Material 1 Research Collective recruitment advertisement.pdf]

SHAPE THE AGENDA

ON THE RESEARCH PRIORITIES FOR WOMEN'S SPORT ON THE ISLAND OF IRELAND*

**INCLUDES EXERCISE AND PHYSICAL ACTIVITY*

Galway Sports Partnership – Comhpháirtíocht Spóirt na Gaillimhe

Donegal Sports Partnership – Sport Ireland

Spórt Éireann Sport Ireland

APPLY TO JOIN THE CO-PRODUCTION COLLECTIVE

Aim: Co-design & develop a research project to drive the research agenda on women's sport.

What's involved:
Attend 2x2hr online meetings & 2hr desk work:

- Read research materials
- Give your perspective on research design & findings

How: Scan the QR code for more information & to express your interest in joining the collective.

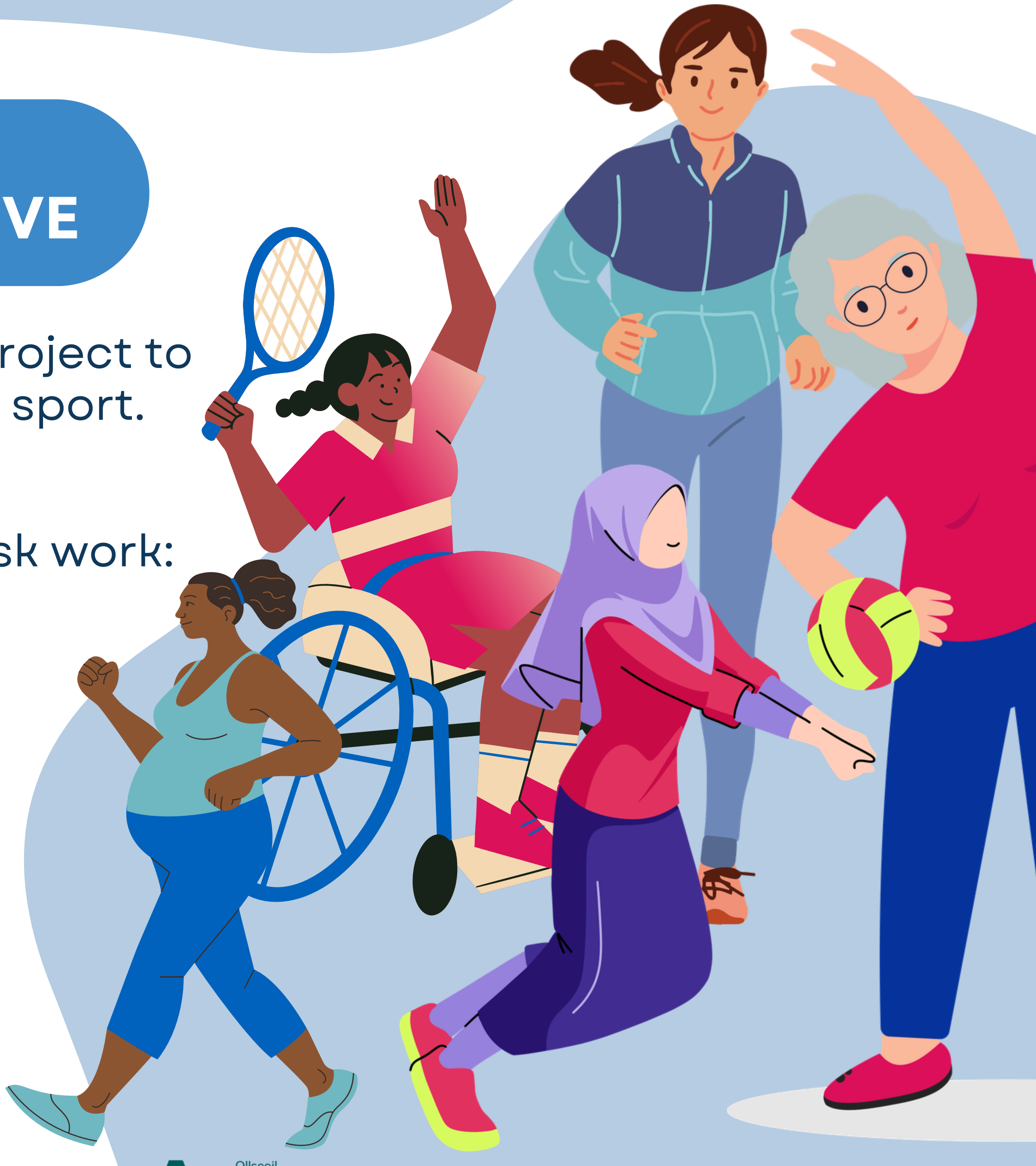

Express interest by **Dec 2nd, 2024**

For more info, email:
Denise.Martin@atu.ie

Ollscoil Teicneolaíochta an Atlantaigh – Atlantic Technological University
Ulster University
TUS
University of Lincoln
