# Supplementary material for: Balancing principles, power, and pragmatism: reflections on co-production in establishing national research priorities for women’s sport and physical activity
Source: Health Promot Int. 2026 Nov 4;41(6):daag159. doi: 10.1093/heapro/daag159 (PMC13732305; doi:10.1093/heapro/daag159)
Supplement: Supplementary file 1 [file daag159_supplementary_data.zip › Supplementary Material 2 Research Collective expression of interest form.pdf]

Women in Sport Research Collective Expression of Interest

Project Background The project is being led by the Local Sport Partnerships in Donegal and Galway, in partnership with Atlantic Technological University, in collaboration with Ulster University, Technological University of the Shannon, and University of Lincoln, UK. Academic partners involved in the project will oversee and lead communication on the project. This research project is a seminal initiative for a new all island research consortium for women and girls in sport on the island of Ireland which will be launched early in 2025.

Project Plan The aim of the Research Collective is to improve research quality by ensuring that the views of women and girls in sport, from diverse backgrounds and settings, and the people who support them, are considered throughout the research process.

What will the Collective Do? The Research Collective will comprise of up 15 people who have experience working with women and girls in different contexts and environments, from different backgrounds and geographical areas. It will involve a commitment of approximately 6-10 hrs of your time between January and June 2025 that will be compensated for, subject to individual and funding requirements.
Members of the Collective will have the following responsibilities:

- Attend 2 online meetings between January and May (1.5 – 2 hours each)
- Before meetings, read the agenda for the meeting
- During meetings, offer a perspective on the development of the research (e.g., a survey), how it will be carried out, who should be recruited and how, and how to put research findings into practice
- Spend time (~2 hours) reviewing the results of a survey and provide your perspective on the findings
- Before and after meetings, contribute via email to requests (e.g., confirmation of minutes)

To balance the different experiences in this group, we invite you to let us know the groups and contexts you are working in now and in previous roles (professional and voluntary) and to confirm your interest in this Collective

1. Please state your current/previous role(s) within women and girls' sport including the role title(s) and organisation(s): *

2. What groups did/do you primarily work with in women and girls' sport? (Please check all that apply) *

- [ ] Predominantly Women / Girls
- [ ] Mixed Gender groups
- [ ] Young Children
- [ ] Adolescents (13-18 years)
- [ ] Adults
- [ ] Older Adults (65+ years)
- [ ] People of different ethnicity
- [ ] People from the LGBTQI+ community
- [ ] People with disabilities
- [ ] People from areas of significant social disadvantage

3. Please tell us some more about the extent of your experience working with these groups in women and girls' sport *(E.g., I worked with XX organisation on a regular basis for 10 years.)*

4. We are interested in the context in which women and girls participate in sport and similar activities.
   Please indicate the various contexts you work or have experience working in by checking all that apply: *

- ☐ Performance sport based (e.g., Provincial Representative Team)
- ☐ Grassroots sport based (e.g., Community football club)
- ☐ Physical activity based (e.g., Community gardening activities)
- ☐ Exercise based (e.g., Pilates classes)
- ☐ Urban based
- ☐ Rural based
- ☐ Mix of rural / urban based experience
- ☐ Northern Ireland based
- ☐ Irish Republic based
- ☐ Based North and South

5. Please add your name *

6. Please add your email address *

7. Please confirm your interest in joining the Research Collective. *

- ◯ Yes, I would like to considered for this position
- ◯ No, I would not like to be considered for this position

8. If no, there will be ways for you to stay connected to the project. If you would like to hear more about the project outcomes, you can consent to do so below. *

- ◯ I consent for my email address to be stored for up to 1 year so the research team can let me know about the findings of the research and any events which are associated with it.

9. Thank you for taking time to express an interest in joining this collective. There is a limit on the number of people who can sit on the group and we will be in touch in the coming weeks to let you know if you have been selected.

   Even if you are not part of the collective, we would like to keep you informed about our findings. Please indicate below if you would like us to keep in touch.

   I consent for my email address to be stored for up to 1 year so the research team can let me know about the findings of the research and any events which are associated with it. *

   ◯ Yes

   ◯ No

This content is neither created nor endorsed by Microsoft. The data you submit will be sent to the form owner.

Microsoft Forms
